# Fatal Panton-Valentine leukocidin-positive MSSA toxic shock syndrome: a call for rapid virulence detection and optimized antimicrobial therapy

**DOI:** 10.3389/fcimb.2026.1805801

**Published:** 2026-05-12

**Authors:** Congcong Zhao, Rongrong Ren, Wei Wu, Jingxian Liu, Chun Chen, Na Yin, Lixia Li

**Affiliations:** 1 Department of Pharmacy, Xin Hua Hospital Affiliated to Shanghai Jiao Tong University School of Medicine, Shanghai, China; 2 Department of Pharmacy, The First People’s Hospital of Xiangtan City, Xiangtan, Hunan, China; 3 Department of Intensive Care, Xin Hua Hospital Affiliated to Shanghai Jiao Tong University School of Medicine, Shanghai, China; 4 College of Pharmacy, Dalian Medical University, Dalian, Liaoning, China; 5 Department of Clinical Laboratory, Xin Hua Hospital Affiliated to Shanghai Jiao Tong University School of Medicine, Shanghai, China

**Keywords:** anti-staphylococcal β-lactams, cefazolin, methicillin-sensitive *Staphylococcus aureus*, Panton-Valentine leukocidin, toxic shock syndrome, vancomycin

## Abstract

**Background:**

Toxic shock syndrome (TSS) is a rare but potentially life-threatening toxin-mediated infection. Cases associated with Panton-Valentine leukocidin (PVL)-producing *Staphylococcus aureus* are particularly uncommon in clinical settings and remain insufficiently documented in the literature.

**Objectives:**

This report aims to strengthen clinical understanding of TSS caused by methicillin-sensitive *Staphylococcus aureus* (MSSA) harboring PVL, assess optimal antimicrobial strategies for treating MSSA infections, minimize the risk of therapeutic failure, and offer insights that may inform timely diagnosis and effective clinical management.

**Methods:**

We present a case involving a 26-year-old female patient with MSSA isolated from multiple anatomical sites who initially received vancomycin before being diagnosed with PVL-positive MSSA-induced TSS. Therapeutic efficacy was evaluated by integrating her clinical course with contemporary evidence on the management of severe MSSA infections. In addition, treatment considerations for highly virulent MSSA infections with distinct clinical characteristics were examined.

**Results:**

The patient’s clinical course enabled a comparative assessment of β-lactam antibiotics, such as cefazolin, versus glycopeptide agents, such as vancomycin, in the management of MSSA infections. Upon confirmation of MSSA, early transition to targeted therapy with cefazolin or anti-staphylococcal penicillins may be considered. In critically ill patients, early virulence gene testing is important for rapid identification of PVL-positive strains.

**Conclusions:**

PVL-positive MSSA may be associated with a fulminant, atypical toxic shock syndrome-like phenotype characterized by disseminated pustulosis and pancytopenia, which should raise immediate suspicion of a toxin-driven process. This case suggests that de-escalation from vancomycin to a β-lactam antibiotic upon MSSA confirmation may be a key therapeutic strategy. Increased vigilance, rapid virulence factor testing, and early aggressive management are important to improving outcomes.

## 1 Introduction

*Staphylococcus aureus* is a prevalent human pathogen classified as methicillin-resistant *Staphylococcus aureus* (MRSA) or methicillin-sensitive *Staphylococcus aureus* (MSSA), depending on methicillin susceptibility. While MRSA has long been prioritized in clinical research because of its multidrug resistance, the pathogenic mechanisms and optimal therapeutic approaches for MSSA require further elucidation, particularly regarding antimicrobial selection and treatment timing (Stevens, 2006; Lodise et al., 2007). MSSA predominantly colonizes the nasal mucosa and skin, and breaches in epithelial or immune defenses may permit invasion via skin disruption or the respiratory tract. In severe circumstances, especially when potent virulence factors are present, infection can progress to toxic shock syndrome (TSS). TSS is an uncommon toxin-mediated illness characterized by sudden onset, fulminant deterioration, and the potential for profound shock and multi-organ dysfunction. Reported mortality ranges from 3% to 22% (Bartlett et al., 2011; AtChade et al., 2024), and approximately 24% of survivors experience lasting sequelae including limb loss or joint dysfunction (Celie et al., 2020). TSS most frequently arises from toxin-producing MSSA or *Streptococcus pyogenes* infections (Wilkins et al., 2017; AtChade et al., 2024). The syndrome was first formally described by James Todd and colleagues in *The Lancet* in 1978 (Todd et al., 1978). Epidemiologic surveillance indicates that the annual incidence of staphylococcal TSS is approximately 0.03–0.07 per 100,000 individuals (Sharma et al., 2018), with the highest incidence observed among women aged 13–24 years, reaching up to 1.41 per 100,000 (DeVries et al., 2011). Here, we describe a case of TSS associated with a Panton-Valentine leukocidin (PVL)- positive MSSA strain, characterized by abrupt clinical deterioration, rapid systemic dissemination, difficulty identifying the primary infectious focus, and atypical cutaneous and hematologic manifestations. The progression of disease in this patient posed substantial diagnostic and therapeutic challenges. By synthesizing current clinical evidence and recent research advances, this report evaluates targeted treatment strategies for highly pathogenic MSSA infections and aims to inform improved diagnostic and management practices for similar clinical scenarios.

This fatal case of TSS associated with PVL-positive MSSA highlights a critical and potentially underappreciated clinical challenge. It exemplifies the peril of diagnostic delay when confronting atypical presentations and underscores the life-threatening consequences of suboptimal antimicrobial selection in MSSA bacteremia, even when guideline-directed empiric therapy is administered. By integrating this case with contemporary evidence, we aim to issue a pressing reminder to clinicians and refine the approach to this highly virulent infection.

## 2 Case presentation

### 2.1 Patient characteristics

A 26-year-old woman weighing 49kg presented with a one-week history of lower back pain radiating to both lower extremities, with marked exacerbation during the preceding 48 hours. Her medical history was notable for a cesarean section five years earlier and an induced abortion two months prior to admission. She denied recent fever, headache, chest or abdominal pain, trauma, or any invasive interventions. The patient denied any recent international travel within the six months preceding admission. On initial examination, her vital signs were as follows: body temperature 36.6°C, heart rate 75 bpm, respiratory rate 18 breaths per minute, and blood pressure 96/56 mmHg.

### 2.2 Major treatment course

**Figure 1:**
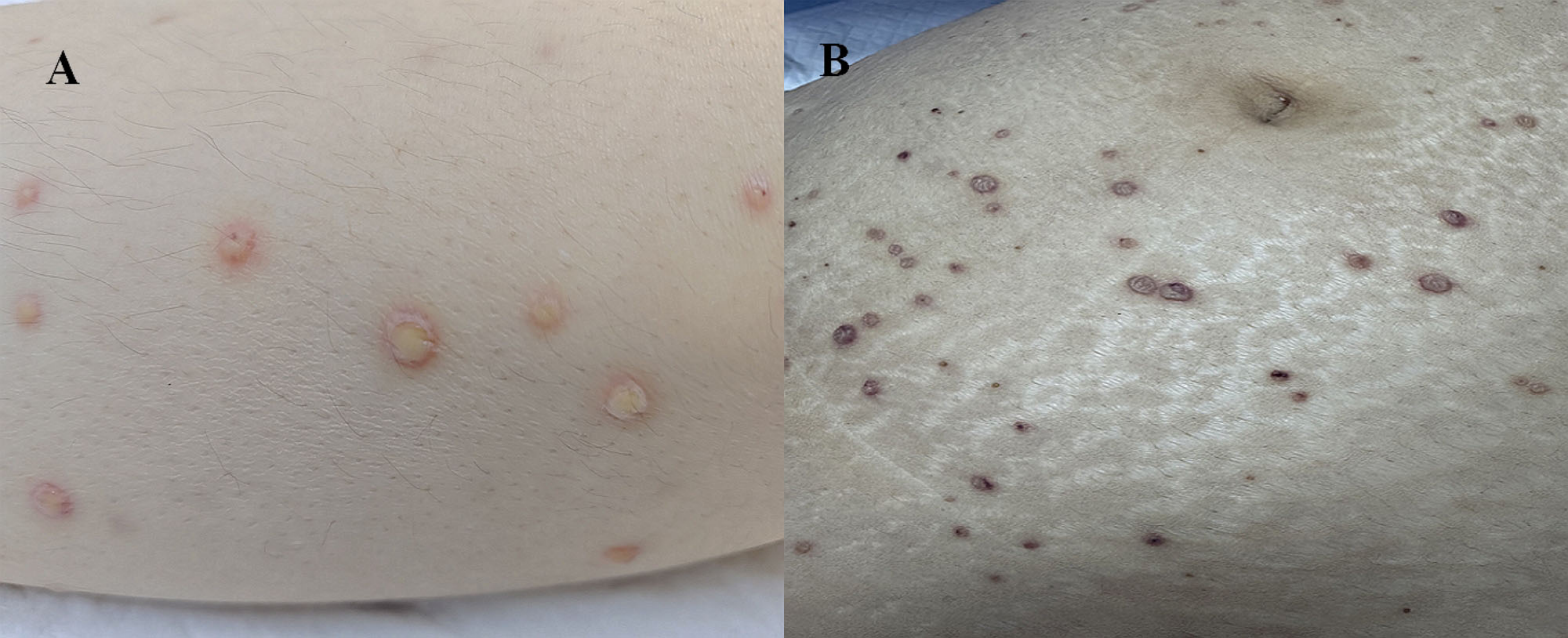
Cutaneous manifestations observed in the patient. **(A)** Scattered erythematous pustules on the lateral aspect of the left thigh. **(B)** Abdomen with scattered erythematous pustules around the periumbilical region.

**Figure 2:**
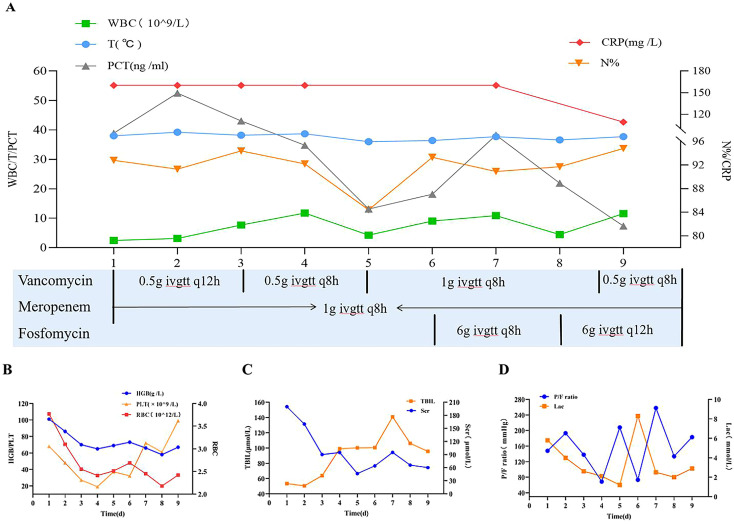
Relevant indicators and medication usage during hospitalization. X-axis represents the time after admission to the Surgical Intensive Care Unit. **(A)** Dynamic changes in key infection parameters during antimicrobial therapy. WBC, white blood cell; T, temperature; PCT, procalcitonin; CRP, C-reactive protein; N, neutrophil. **(B)**Temporal trends in hematologic parameters during treatment. HGB, hemoglobin; RBC, red blood cell count; PLT, platelet count. **(C)** Temporal trends in hepato-renal function during treatment. Scr, serum creatinine; TBIL, total bilirubin. **(D)** Dynamics of Oxygen Delivery-Hypoxia Balance. P/F ratio, ratio of arterial oxygen partial pressure to fraction of inspired oxygen; Lac, lactate; ivgtt, intravenous guttae.

On the second hospital day, the patient experienced sudden onset of severe pain in all extremities accompanied by dyspnea. This was followed by rapidly progressive generalized pain and profound weakness. Her vital signs deteriorated, with a body temperature of 38 C, heart rate of 160 bpm, respiratory rate of 32 breaths per minute, and blood pressure of 89/45 mmHg. Marked tenderness was noted across the extremities and trunk, along with scattered erythematous pustules on the lower extremities and trunk (**Figure 1A**). She was immediately transferred to the surgical intensive care unit (SICU). Key clinical parameters throughout hospitalization are summarized in **Figure 2**. Chest radiography revealed bilateral pulmonary infiltrates with suspected pleural effusions. Empiric antimicrobial therapy was initiated with vancomycin plus meropenem, with dosing details provided in **Figure 2A**.

On SICU Day 2, the patient required continued mechanical ventilation via endotracheal tube. She remained febrile and hemodynamically unstable, with blood pressure of 114/42 mmHg maintained using vasoactive support and a heart rate of 144 bpm. Diffuse pustules were observed across the body (**Figure 1B**), accompanied by pancytopenia and elevated inflammatory biomarkers. Intravenous immunoglobulin (IVIG) was initiated at 20g daily.

**Figure 3:**
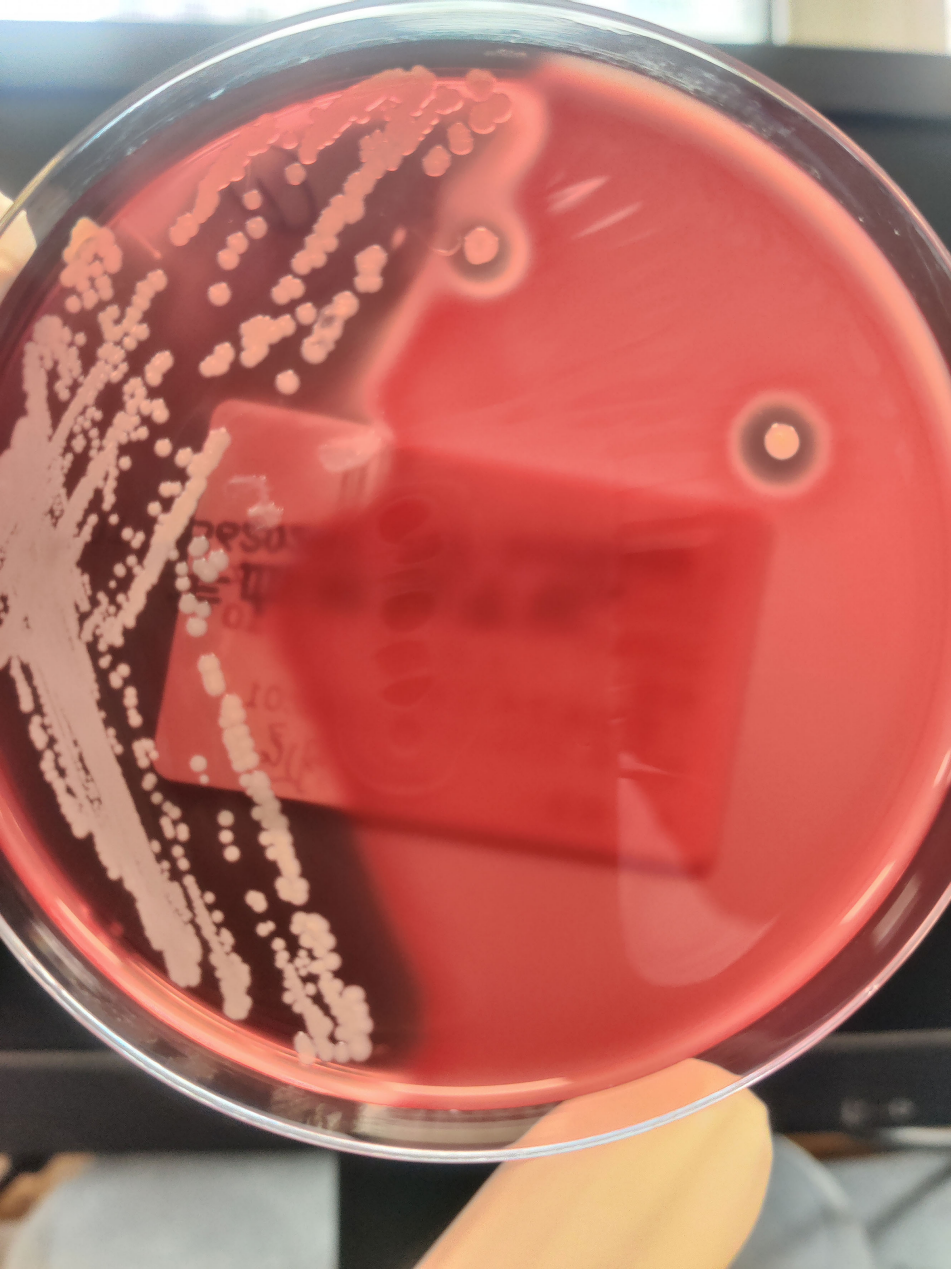
Colony morphology of MSSA isolated from sputum culture on blood agar. MSSA colonies on blood agar showing a large hemolytic zone diameter. It suggests that this MSSA isolate exhibits high hemolysin production. Hemolysins are critical virulence factors in staphylococci that disrupt host tissue barriers, promote bacterial dissemination, and lyse immune cells. The observed phenotype indicates that this isolate possesses potentially heightened virulence.

On SICU Day 3, blood pressure improved to 138/67 mmHg with a heart rate of 123 bpm. Sputum culture yielded MSSA, with antimicrobial susceptibility results presented in **Table 1**, and colony morphology shown in **Figure 3**. Chest radiography revealed worsening bilateral pulmonary exudates and suspected pleural effusion, prompting the performance of ultrasound-guided thoracentesis and drainage. Consequently, the dose of vancomycin was further increased.

**Table 1:** Results of antimicrobial susceptibility testing for MSSA.

Antimicrobial agents	Day 3 sputum culture		Day 4 sputum/blood/urine culture		Day 7 leg pus/abdominal pus/sputum culture	
	MIC (mg/L)	Susceptibility	MIC (mg/L)	Susceptibility	MIC (mg/L)	Susceptibility
Penicillin G	≥ 0.5	R	≥ 0.5	R	≥ 0.5	R
Oxacillin	≤ 0.25	S	≤ 0.25	S	0.5 (abdominal pus) ≤ 0.25 (leg pus/sputum)	S
Ceftaroline	0.25	S	0.25	S	0.25	S
Gentamicin	≤ 0.5	S	≤ 0.5	S	≤ 0.5	S
Rifampicin	≤ 0.5	S	≤ 0.5	S	≤ 0.5	S
Levofloxacin	0.25	S	0.25	S	0.25	S
Moxifloxacin	≤ 0.25	S	≤ 0.25	S	≤ 0.25	S
Co-trimoxazole	≤ 10	S	≤ 10	S	≤ 10	S
Clindamycin	0.25	S	27 (urine, KB method) 0.25 (sputum/blood, MIC method)	S	0.25	S
Daptomycin	0.25	S	0.25	S	0.25	S
Erythromycin	≤ 0.25	S	27 (urine, KB method) ≤ 0.25 (sputum/blood, MIC method)	S	≤0.25	S
Linezolid	2	S	2	S	2	S
Vancomycin	1	S	≤ 0.5 (urine) 1 (sputum/blood)	S	1 (leg pus/sputum) ≤ 0.5 (leg pus)	S
Teicoplanin	≤ 0.5	S	≤ 0.5	S	≤ 0.5	S
β-lactamase	Positive	+	Positive	+	Positive	+

MIC, Minimum Inhibitory Concentration; R, resistant; S, susceptible; KB method, Kirby-Bauer disk diffusion method.

**Figure 4:**
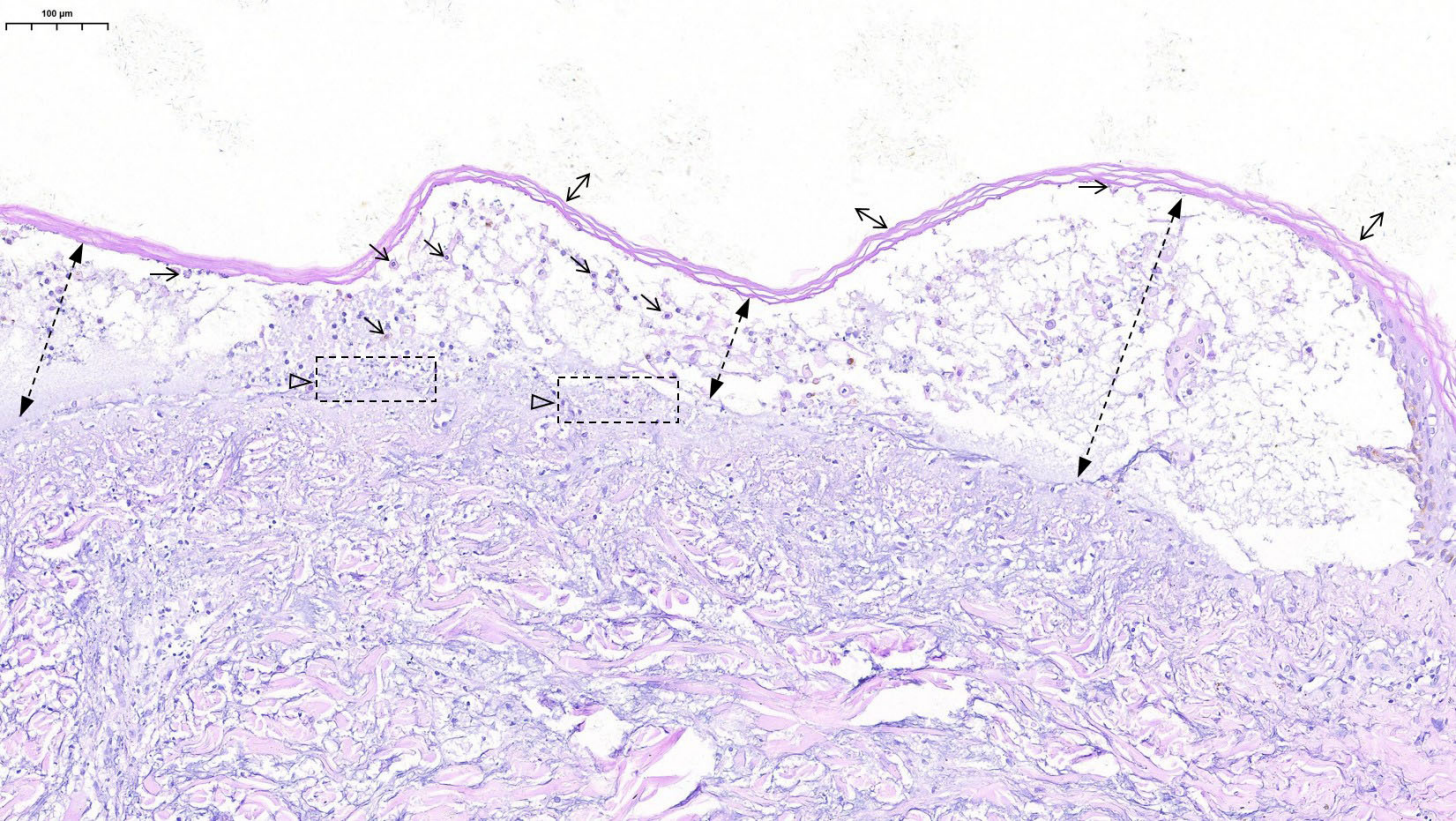
Histopathological examination of the skin lesion (hematoxylin and eosin stain; scale bar = 100 μm). Key pathological features are indicated as follows: bidirectional arrow (↔), hyperkeratosis; dashed line with solid arrowhead (▶), focal dermo-epidermal separation; horizontal arrow (→), degenerated keratinocytes within the epidermis; oblique arrow (↘), acantholytic and edematous keratinocytes within the blister cavity; open arrowhead (▷) with dashed frame, lymphocytic infiltration with nuclear dust in the underlying dermis. Additional findings include wedge-shaped degeneration and obscuration of small vessel architecture.

On SICU Day 4, chest radiography again demonstrated diffuse exudates suggestive of pulmonary edema. A sudden drop in oxygen saturation to 84% prompted initiation of continuous renal replacement therapy. A bedside chest radiograph confirmed left-sided pneumothorax, and thoracentesis was performed for air evacuation. Blood, urine, and sputum cultures all tested positive for MSSA (**Table 1**). Histopathologic examination of the skin lesion is presented in **Figure 4**. A multidisciplinary consultation was undertaken to consider a transition to cefazolin-based therapy, although a consensus recommendation was not achieved.

On SICU Day 5, multiplex testing for respiratory pathogens identified only *Staphylococcus aureus* DNA, while results for influenza A and B viruses, other respiratory viruses, and common bacterial pathogens were negative. Imaging again showed left-sided pneumothorax with bilateral pneumonia, and vancomycin dosing was escalated. On SICU Day 6, vancomycin trough and peak concentrations measured at 9.56 µg/mL and 19.54 µg/mL, respectively, and adjunctive fosfomycin was introduced.

**Figure 5:**
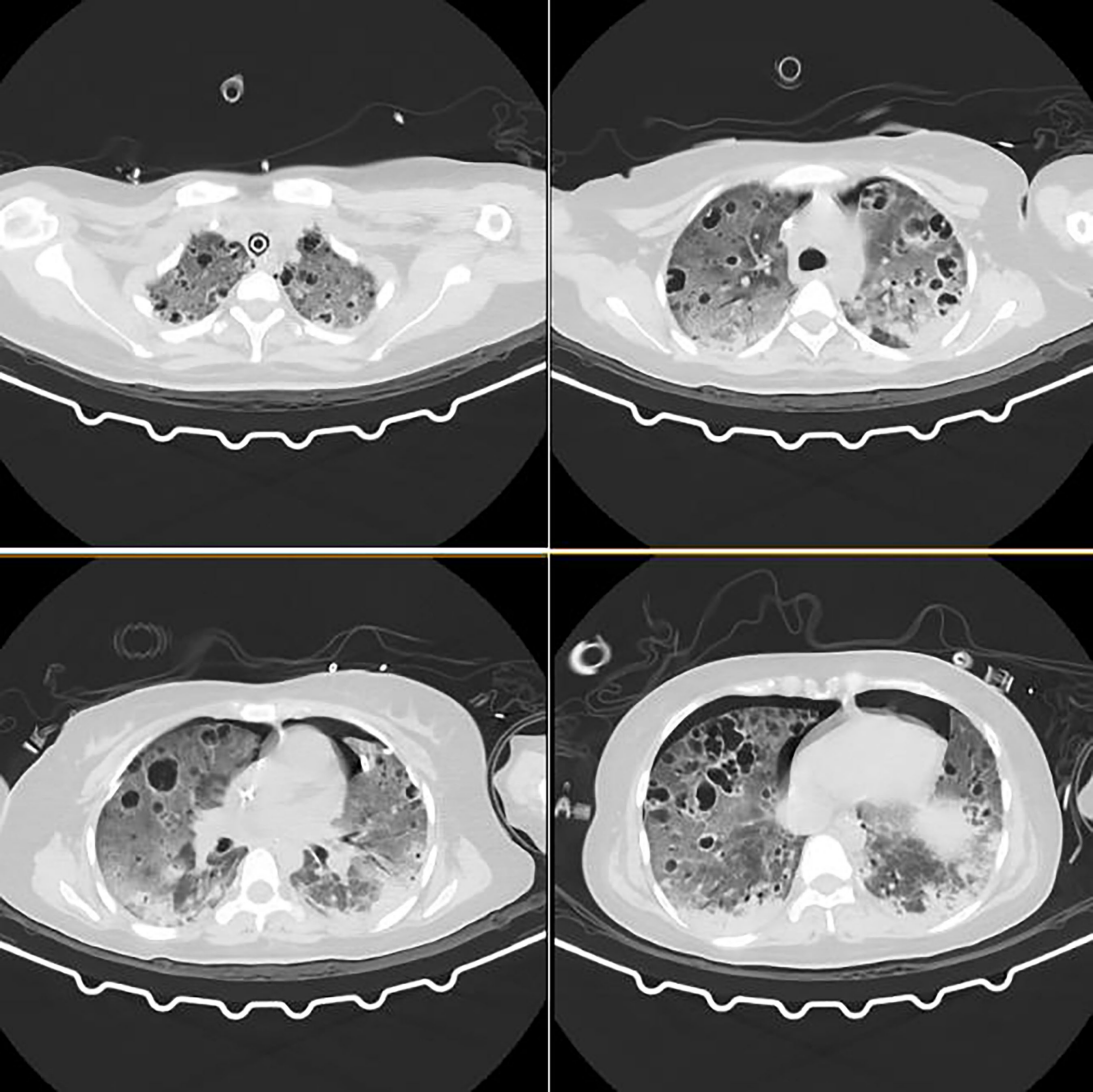
Chest computed tomography imaging on day 7 in the SICU. A chest CT scan demonstrated diffuse pulmonary cystic lesions with ground-glass opacities, multiple solid nodules bilaterally, and pleural effusion with consolidation and atelectasis in both lower lobes.

On SICU Day 7, pus and sputum cultures remained MSSA positive. Computed tomography of the chest revealed diffuse cystic changes throughout both lungs (**Figure 5**). On SICU Day 8, serum sodium rose to 161 mmol/L, necessitating a reduction in fosfomycin dosage.

On SICU Day 9, the patient continued to require mechanical ventilation and chest tube drainage. The targeted next generation sequencing results (see Section 2.3) confirmed the PVL-positive status of the MSSA isolate. Given the severe and progressively deteriorating clinical course, the family elected to withdraw care and proceed with voluntary discharge.

### 2.3 Microbiological investigation

Sputum, blood, urine, and pus specimens were collected at specified time points (Days 3–7) and sent to the clinical microbiology laboratory for culture and antimicrobial susceptibility testing (AST). AST was performed using the AST-639 card on the VITEK 2 Compact automated susceptibility system (bioMérieux, Marcy-l’Étoile, France), with results interpreted in accordance with the Clinical and Laboratory Standards Institute (CLSI) M100 guidelines (2025 edition). The *mecA* gene was detected using Loop-mediated isothermal amplification (LAMP) method on the RTisohip-A Nucleic Acid Analyzer (CapitalBio Technology Inc., Beijing, China), and was found to be negative, confirming the isolate as MSSA. The targeted next generation sequencing confirmed the presence of both *lukS-PV* and *lukF-PV* genes, which encode the two subunits of PVL. The minimum inhibitory concentration (MIC) values for key antimicrobial agents are summarized in **Table 1**.

It is noteworthy that the cefazolin MIC was not directly tested, in line with CLSI selective reporting guidelines. Given that the isolate was oxacillin-susceptible (MIC ≤ 0.25 μg/mL), susceptibility to cefazolin can be reliably inferred. Ceftaroline (a fifth-generation cephalosporin) was tested and demonstrated an MIC of 0.25 μg/mL, indicating susceptibility.

### 2.4 Molecular typing

Multilocus sequence typing (MLST) was conducted on the clinical isolate of MSSA. The results indicated that the strain belongs to sequence type 22 (ST22). Recently, ST22 has emerged as a predominant clone of Panton-Valentine leukocidin (PVL)-positive MSSA in several regions of China, including Beijing and Urumqi (Xiao et al., 2019; Yuan et al., 2019).

## 3 Discussion

### 3.1 The diagnostic challenge of staphylococcal TSS without classical desquamation

The diagnostic criteria for staphylococcal TSS were first defined by the Centers for Disease Control and Prevention (CDC) in the 1980s and later updated in 2011 (AtChade et al., 2024). Typical clinical manifestations include abrupt onset of high fever, diffuse rash, hypotension, and dysfunction involving three or more organ systems. A definitive diagnosis requires desquamation, which typically occurs 1–2 weeks after symptom onset. Because desquamation of the palms and soles generally appears 8 to 21 days after the onset of illness, the CDC criteria predominantly support retrospective confirmation. During the acute phase, patients may fail to meet all criteria, and strict reliance on these guidelines can delay urgent therapeutic intervention (Berger et al., 2019).

In the present case, the patient initially reported lower back pain, followed by high fever, hypotension, multiple infectious foci including bloodstream infection, skin and soft tissue involvement, necrotizing pneumonia, and evolving multi-organ dysfunction. Cutaneous findings consisted of scattered small pustules that first appeared on the extremities and rapidly disseminated across the trunk and to the head and neck, consistent with clinical diagnostic criteria for staphylococcal TSS (AtChade et al., 2024). Given that the patient tested negative for influenza and other respiratory viruses, the back and muscle pain are not considered to be symptoms related to influenza. It is noteworthy that the clinical overlap between TSS and sepsis frequently results in delayed recognition and treatment.

Therefore, a high index of suspicion, based on a constellation of severe and rapidly progressive symptoms—even in the absence of classic desquamation—should take precedence over strict adherence to the CDC criteria during acute management. Approximately 40% of non-menstrual TSS cases do not present with an identifiable primary infectious focus (DeVries et al., 2011). Diagnoses become further complicated when TSS arises in conjunction with surgical procedures or traumatic injuries, which may exhibit prolonged latency periods (Celie et al., 2020). In the present case, the initial focus of infection was unclear at the time of admission. The patient’s recent history of abortion two months prior may represent a relevant predisposing factor that facilitated MSSA colonization and subsequent toxin-mediated disease. These findings suggest that postoperative staphylococcal TSS may have a particularly prolonged latency period, necessitating sustained clinical vigilance.

### 3.2 Atypical clinical features of PVL-associated TSS: pancytopenia and extreme PCT elevation

Although thrombocytopenia is a recognized diagnostic parameter of staphylococcal TSS (AtChade et al., 2024), most reported cases demonstrate leukocytosis and neutrophilia during the acute stage (Berger et al., 2019). Conversely, this patient exhibited pancytopenia, a phenomenon likely attributable to PVL-mediated polymorphonuclear neutrophil lysis. Furthermore, serum procalcitonin (PCT) concentrations are generally higher in gram-negative bacteremia than in gram-positive infections (Thomas-Rüddel et al., 2018), and levels in staphylococcal disease are usually lower than those observed in streptococcal infections (Nejtek et al., 2024). However, this patient demonstrated a peak PCT value of 52.25 ng/mL, indicating a profound toxin-driven inflammatory response consistent with severe TSS.

The cutaneous manifestations in this case warrant specific attention. The patient developed disseminated pustular lesions that rapidly spread from the extremities to the trunk and head, diverging from the more typical diffuse erythroderma described in classical TSS. This pustular phenotype has been documented in certain PVL-positive staphylococcal infections and may serve as an early clinical indicator of the presence of this toxin. Notably, there was no reported history of skin and soft tissue infections, particularly skin abscesses, in the patient or their close contacts. Consequently, based on this clinical information, intrafamilial transmission of PVL-*S. aureus* is considered unlikely. The cutaneous pustular lesions may reflect either the entry site for MSSA or secondary hematogenous dissemination, underscoring the inherent challenges in determining the infection source in cases of TSS.

In summary, the patient exhibited clinical characteristics divergent from classical staphylococcal TSS, including the abrupt onset of disseminated pustular rash, extreme PCT elevation, pancytopenia, and the absence of an identifiable primary focus. These features warrant heightened clinical suspicion for PVL-positive staphylococcal infection and underscore the importance of early recognition to avoid delays in targeted management. The disseminated pustulosis and pancytopenia observed in our patient served as significant, albeit atypical, indicators of a PVL-positive staphylococcal infection associated with TSS-like features.

### 3.3 The controversial role of PVL in toxic shock syndrome

TSS is primarily attributed to toxic shock syndrome toxin-1 (TSST-1) and staphylococcal enterotoxins A, B, and C (SEA, SEB, SEC) (AtChade et al., 2024). Our sequencing panel analyzed 64 virulence genes, including these critical TSS-associated toxins, none of which were detected. Consequently, this comprehensive genetic analysis eliminates the possibility that the MSSA strain produces other superantigenic enterotoxins as alternative etiological factors for TSS, with the exception of PVL.

Although PVL is a well-recognized virulence factor associated with skin and soft tissue infections as well as necrotizing pneumonia, its specific role in the pathogenesis of toxic shock syndrome—particularly in the absence of classical superantigens such as TSST-1 or staphylococcal enterotoxins—remains incompletely defined. Recent evidence suggests that PVL-positive, superantigen-negative *Staphylococcus aureus* strains can, albeit rarely, be associated with a fulminant toxic shock-like phenotype (Reichert et al., 2005; Cuddihy et al., 2021; Zhang et al., 2022; Attou et al., 2026). However, most published cases of PVL-associated toxic shock syndrome have also harbored additional toxin genes, including TSST-1 and/or staphylococcal enterotoxins (Mushtaq et al., 2008; Li et al., 2011; Hayakawa et al., 2020). A large multicenter study from Ethiopia reported a significant association between the presence of the TSST-1 gene and the pvl gene (P < 0.001) (Ibrahim et al., 2023). Furthermore, the role of PVL in systemic inflammatory syndromes remains a subject of controversy. Large-scale meta-analyses have indicated that PVL genes are relatively rare in cases of bacteremia (Shallcross et al., 2013). Additionally, studies utilizing animal models have demonstrated that PVL-knockout strains exhibit virulence comparable to that of wild-type strains in mouse sepsis models, suggesting that PVL may not serve as the primary virulence determinant in invasive diseases (Voyich et al., 2006).

While the clinical and microbiological features of our case suggest a potential contributory role of PVL in the development of toxic shock syndrome, a causal relationship cannot be definitively established based on a single case report. We acknowledge that other known or unidentified virulence determinants may have also contributed to disease pathogenesis. Therefore, our findings should be interpreted as hypotheses rather than definitive evidence. Future research incorporating whole-genome sequencing and functional toxin assays is essential to elucidate the specific role of PVL in the pathogenesis of TSS.

### 3.4 The virulence of PVL and imperative for rapid testing

PVL is a pore-forming exotoxin that selectively targets polymorphonuclear neutrophils (PMNs) and is commonly associated with necrotizing pneumonia (Rájová et al., 2016). Upon binding to PMNs, PVL triggers chemotactic recruitment followed by rapid cell lysis, resulting in the release of cytotoxic enzymes and oxidants that drive extensive pulmonary tissue destruction and can lead to fulminant and often fatal necrotizing pneumonia (Diep et al., 2010). PVL-associated necrotizing pneumonia is characterized by an exceedingly rapid clinical course (Diep et al., 2013). Most affected patients initially present with sepsis or septic shock, typically in the context of community-acquired *S. aureus* infection. These observations emphasize that in previously healthy individuals experiencing rapidly progressive community-onset *S. aureus* disease, early assessment for virulence genes through molecular testing of blood or other infected specimens is imperative.

In critically ill patients suffering from community-onset fulminant *Staphylococcus aureus* infections, waiting for microbiological confirmation of PVL is a luxury that clinicians cannot afford. Therefore, molecular testing should be pursued aggressively and empirically at the earliest suspicion of infection. In the absence of molecular testing, rapid lateral flow assays provide a practical and cost-effective method for preliminary screening, facilitating earlier clinical intervention.

### 3.5 Molecular epidemiology of PVL-positive MSSA in China

To provide epidemiological context for this case, we reviewed the existing literature on PVL-positive MSSA in China. The prevalence of PVL-positive Staphylococcus aureus among clinical isolates in China has been reported to be approximately 7.2%. Notably, the carriage rates of PVL are generally higher in MSSA than in MRSA isolates; a national survey of 809 isolates from 18 teaching hospitals found PVL carriage in 11.1% of MSSA and 3.5% of MRSA isolates (Du et al., 2008). Regarding clonal distribution, molecular typing studies have revealed diverse clonal complexes (CCs) among PVL-positive MSSA in China. In a prospective study of skin and soft tissue infections conducted in Beijing from 2009 to 2010, the most prevalent MSSA clones were ST398 (17.6%), ST7, and ST1, with an overall PVL positivity rate of 41.5% among MSSA isolates (66 out of 159) (Zhao et al., 2012). Notably, a high prevalence of the livestock-associated ST398 clone (17.1% of all *Staphylococcus aureus* infections) was found, with no apparent association with animal contact (Zhao et al., 2012). More recently, a genomic epidemiology study of invasive bloodstream isolates from 17 Chinese provinces conducted between 2014 and 2019 identified CC188 (17.14%), CC398 (15.71%), and CC5 (15.71%) as the most frequent clonal complexes among MSSA, with 6 of the 175 sequenced strains carrying PVL genes (Jin et al., 2022). Importantly, a study from Urumqi reported that ST22-MSSA-t309 was the most common MSSA clone (26.0%, 87 out of 334), and 80.8% of ST22-MSSA strains harbored the pvl gene, with the majority (87.5%) isolated from abscesses or wounds (Yuan et al., 2019).

Our isolate was identified as sequence type 22 (ST22), a predominant clone of PVL-positive methicillin-sensitive Staphylococcus aureus (MSSA) that has recently emerged in China. In Beijing, Xiao et al. reported that ST22 accounted for 48.6% of MSSA isolates from skin and soft tissue infections in adults, with a PVL positivity rate of 50% (Xiao et al., 2019). To our knowledge, this is the first report of a fatal TSS case caused by PVL-positive ST22 MSSA, thereby extending the clinical spectrum of this emerging clone beyond the commonly reported skin and soft tissue infections to include fulminant toxin-mediated systemic disease. Collectively, these data indicate that PVL-positive MSSA is not uncommon in China, exhibiting diverse clonal backgrounds and a predilection for community-onset infections. While we acknowledge the absence of whole-genome sequencing as a limitation, our MLST result (ST22) and the contextual evidence underscore the clinical relevance of recognizing PVL-positive MSSA as a potential cause of fulminant TSS in China.

### 3.6 Antimicrobial selection: A pivotal determinant of outcome

The recommended first-line antimicrobial regimen for MSSA-associated TSS is oxacillin or nafcillin in combination with clindamycin, with cefazolin plus clindamycin as an accepted alternative (Bartlett et al., 2011; Gilbert et al., 2023). Clindamycin is administered to inhibit bacterial protein synthesis, thereby suppressing exotoxin production and mitigating toxemic manifestations. Linezolid exerts a similar inhibitory effect on toxin synthesis (Gilbert et al., 2023), and both clindamycin and linezolid have demonstrated mortality reduction and improved clinical outcomes in TSS (Babiker et al., 2021; Cortés-Penfield and Ryder, 2023). Current guidelines further recommend cefazolin or other anti-staphylococcal penicillins as the preferred therapeutic agents for MSSA bacteremia (Delgado et al., 2023; Gilbert et al., 2023; McDonald et al., 2023). Vancomycin, although widely used empirically, is not considered a first-line agent for confirmed MSSA infections. Comparative evidence consistently shows that anti-staphylococcal β-lactams such as oxacillin and cefazolin achieve superior bactericidal activity, higher cure rates, and significantly lower mortality in MSSA bloodstream infections (Stryjewski et al., 2007; Chan et al., 2012; McDanel et al., 2015). Mortality associated with vancomycin therapy in MSSA bacteremia has been reported to be two to three times higher than that associated with β-lactams (McConeghy et al., 2013). Several pharmacologic and microbiologic mechanisms contribute to this disparity. Vancomycin exhibits slower bactericidal kinetics and a delayed clearance of MSSA. An elevated minimum bactericidal concentration to minimum inhibitory concentration (MBC/MIC) ratio further compromises its killing efficiency (Stevens, 2006), and certain MSSA strains possess genetic adaptations that reduce vancomycin susceptibility (Kang et al., 2023). Moreover, its large molecular mass (1450 Daltons) hinders adequate penetration through the thick peptidoglycan cell wall of gram-positive organisms, leading to sequestration within the cell wall compartment and reduction of effective intracellular activity (Baquero and Levin, 2021). These mechanistic limitations have been confirmed in multiple *in vitro* studies (Larsson et al., 1996; Cui et al., 2006).

The window for optimal therapy is narrow. In our case, the persistence of vancomycin, despite the confirmation of MSSA, likely contributed to the uncontrolled dissemination of infection and toxin-mediated injury. This case serves as a crucial reminder that in confirmed MSSA infections, vancomycin should not be regarded as a default treatment but rather as a suboptimal agent that should be actively replaced.

Early modification of antimicrobial therapy improves prognosis. Transitioning from vancomycin to nafcillin or cefazolin within the initial treatment phase in MSSA bacteremia has been associated with up to 69% reduction in mortality (Schweizer et al., 2011), while also decreasing complications such as persistent bacteremia and nephrotoxicity (Eby et al., 2018; Austin et al., 2020). Therefore, in the absence of a confirmed β-lactam allergy or access limitations, anti-staphylococcal β-lactams should be prioritized over vancomycin for targeted therapy. In the current case, empiric initiation of vancomycin was appropriate prior to pathogen identification to ensure coverage of possible MRSA infection. Nonetheless, the lack of timely transition to targeted β-lactam therapy following confirmation of MSSA may have contributed to the unsatisfactory clinical outcome.

The optimal treatment duration for TSS remains undefined; however, literature suggests a median course of approximately 12 days (Suzuki et al., 2022). Given the high-risk profile and complexity of MSSA bacteremia, a prolonged therapy duration of three weeks or longer is generally recommended.

The selection between cefazolin and anti-staphylococcal penicillins for the treatment of MSSA infections remains an area of active clinical discussion. Approximately 22% of MSSA isolates demonstrate the cefazolin inoculum effect (CIE), which may reduce cefazolin efficacy in the setting of high bacterial burden (Lee et al., 2018). Despite this concern, multiple studies indicate that cefazolin and anti-staphylococcal penicillins exhibit comparable effectiveness in MSSA bacteremia, while cefazolin confers a lower risk of nephrotoxicity (Monogue et al., 2018; Shi et al., 2018; Hess et al., 2023). When considering combination antimicrobial therapy, treatment decisions should be individualized based on the site of infection and disease severity. Routine combination therapy in uncomplicated MSSA bacteremia is not recommended, as clinical trials have not demonstrated improved outcomes and have shown increased drug-related adverse effects (Tong et al., 2025). For persistent MSSA bacteremia, fosfomycin may be employed as an adjunctive agent. However, current evidence remains limited and inconsistent, with some studies suggesting no clear clinical benefit and an increased incidence of adverse events associated with combination regimens (Pujol et al., 2021; Grillo et al., 2023). Given the rarity of MSSA-induced TSS, optimal combination strategies specific to this severe phenotype remain insufficiently established. In the present case, fosfomycin was administered as adjunctive therapy for disseminated MSSA infection but did not produce a sufficient clinical response.

### 3.7 Adjunctive therapy: the unanswered question of adequate dosing

Management of staphylococcal TSS requires prompt and comprehensive supportive care, including fluid resuscitation, aggressive source control through removal or debridement of infected foci, and antitoxin-directed interventions such as intravenous immunoglobulin (IVIG). Given the central role of toxin-mediated pathology, suppression and neutralization of virulence factors are essential. Patients with TSS frequently exhibit insufficient endogenous antitoxin antibodies. IVIG provides polyclonal antibodies with potential anti-inflammatory and immunomodulatory functions, and *in vitro* evidence confirms its ability to neutralize PVL (Gauduchon et al., 2004). However, significant PVL antibody titers appear to develop only in individuals infected with PVL-positive strains (Croze et al., 2009), raising uncertainty regarding whether commercially available IVIG preparations consistently contain sufficient concentrations of PVL-neutralizing antibodies to exert meaningful clinical effects *in vivo*.

Current guidance varies regarding IVIG dosing. The Johns Hopkins ABX Guide recommends 2 g/kg on day 1, with a second dose if hemodynamic instability persists within 48 hours (Bartlett et al., 2011). The Sanford Guide to Antimicrobial Therapy suggests 1 g/kg on day 1 followed by 0.5 g/kg on days 2 and 3 (Gilbert et al., 2023). Despite these recommendations, evidence supporting mortality reduction from IVIG therapy in TSS remains inconclusive (Parks et al., 2018; Senda et al., 2023). In this case, the patient received IVIG at 20 g/day for five consecutive days, a regimen considerably lower than guideline-recommended dosing, and no significant clinical improvement was observed. This outcome suggests that insufficient IVIG dosing may be inadequate to neutralize PVL or attenuate toxin-mediated systemic injury. Considering that the cumulative dose administered was markedly below guideline recommendations, the absence of clinical response presents a compelling hypothesis: conventional IVIG dosing may be insufficient to neutralize the substantial toxin load associated with fulminant PVL-associated TSS. This observation necessitates further pharmacokinetic and pharmacodynamic studies. In the meantime, it suggests that the early administration of high-dose IVIG should be strongly considered in similar clinical scenarios.

Beyond IVIG, several emerging antitoxin strategies targeting PVL are under evaluation. Experimental agents such as ascorbic acid, nicotinamide, neutralizing monoclonal antibodies, and C5a receptor (C5aR) agonists have demonstrated inhibitory effects on PVL-induced cytotoxicity *in vitro* (Rouha et al., 2018; AlSaleh et al., 2023; Grebe et al., 2024). While promising, these therapeutic approaches require further investigation to determine their efficacy, safety, and clinical applicability in patients with PVL-associated TSS.

## 4 Limitations

First, as a case report, our findings are primarily descriptive, and causal inferences are inherently limited. The decision to withdraw care was influenced by multiple factors, making it impossible to definitively attribute the outcome to any single therapeutic decision. Nevertheless, the compelling temporal progression and substantial supporting literature coalesce to create a powerful narrative that highlights critical, actionable lessons in the management of this lethal syndrome.

Second, while our case indicates an association between PVL production and the development of TSS, a causal relationship has not been firmly established. The isolate tested negative for TSST-1 and the classical staphylococcal enterotoxins (SEA, SEB, SEC), which are the toxins most commonly linked to staphylococcal TSS. Other known or unidentified virulence factors, including additional superantigens, exfoliative toxins, or synergistic interactions among multiple toxins, may have contributed to the severe clinical course. Furthermore, the role of PVL in systemic inflammatory syndromes remains a subject of debate, as large-scale meta-analyses have reported that PVL genes are relatively rare in cases of bacteremia, and animal models suggest that PVL is not the primary virulence factor in invasive diseases. Therefore, our findings should be interpreted as generating hypotheses rather than being conclusive. Future research that incorporates whole-genome sequencing and functional toxin assays is essential to clarify the specific role of PVL in TSS pathogenesis.

Third, we acknowledge the absence of whole-genome sequencing for our isolate as a technical limitation. Additionally, this study represents a single-center experience, and further multicenter studies are necessary to validate our observations.

## 5 Conclusion

This report describes a rare case of TSS associated with PVL-positive MSSA, defined by sudden onset, rapid clinical deterioration, difficulty identifying the infectious source, prolonged incubation relative to preceding gynecologic intervention, pancytopenia, widespread pustular eruption, and a pronounced hyperinflammatory state. The severity and atypical combination of these features underscore the importance of clinician awareness regarding this uncommon presentation. Based on the observed clinical course, we suggest that once MSSA infection is confirmed, a prompt transition to targeted therapy with cefazolin or anti-staphylococcal penicillins may be beneficial. In critically ill patients, early testing for virulence genes and timely incorporation of antitoxin-directed therapies, including antitoxin antimicrobial agents and high-dose IVIG, may serve as valuable adjuncts. The diagnostic and therapeutic experience gained from this case may help refine management strategies for this severe TSS phenotype. However, given the hypothesis-generating nature of our findings, further studies are necessary to establish definitive recommendations.

## Data Availability

The raw data supporting the conclusions of this article will be made available by the authors, without undue reservation.
